# Correlations of *FRMD7* gene mutations with ocular oscillations

**DOI:** 10.1038/s41598-022-14144-7

**Published:** 2022-06-15

**Authors:** Lijuan Huang, Yunyu Zhou, Wencong Chen, Ping Lin, Yan Xie, Kaiwen He, Shasha Zhang, Yuyu Wu, Ningdong Li

**Affiliations:** 1 Department of Ophthalmology, The Second Affiliated Hospital of Fujian Medical University, Quanzhou, 362000 China; 2 Department of Ophthalmology, Beijing Children’s Hospital, Capital Medical University, No 56. Nan Li Shi Rd, Xicheng District, Beijing, 100045 China; 3 Department of Biostatistics, Vanderbilt University Medical Center, 2525 West End Avenue, Suite 1100, Nashville, TN 37203 USA; 4 Department of Ophthalmology, Xi’an Children’s Hospital, Xi’an, 710002 China; 5 Key Laboratory of Major Diseases in Children, Ministry of Education, Beijing, 100045 China; 6 Department of Ophthalmology, Children’s Hospital, Capital Institute of Pediatrics, Beijing, 100020 China

**Keywords:** Mutation, Eye diseases

## Abstract

Mutations in the FERM domain containing 7 (*FRMD7*) gene have been proven to be responsible for infantile nystagmus (IN). The purpose of this study is to investigate *FRMD7* gene mutations in patients with IN, and to evaluate the nystagmus intensity among patients with and without *FRMD7* mutations. The affected males were subdivided into three groups according to whether or not having *FRMD7* mutations and the types of mutations. Fifty-two mutations were detected in *FRMD7* in 56 pedigrees and 34 sporadic patients with IN, including 28 novel and 24 previous reported mutations. The novel identified mutations further expand the spectrum of *FRMD7* mutations. The parameters of nystagmus intensity and the patients’ best corrected visual acuity were not statistically different among the patients with and without identified *FRMD7* mutations, and also not different among patients with different mutant types. The FERM-C domain, whose amino acids are encoded by exons 7, 8 and 9, could be the harbor region for most mutations. Loss-of-function is suggested to be the common molecular mechanism for the X-linked infantile nystagmus.

## Introduction

Infantile nystagmus (IN) is characterized as an involuntary, rhythmic, to-and-for ocular oscillations after early birth, without any disorders in the eyeball or visual pathway^1,2^. It can be inherited in an autosomal dominant or an X-linked pattern, of which the X-linked inheritance pattern is more common, accounting for nearly 90% of cases. In the X-linked pattern, a few females carrying with gene mutation could have nystagmus, but a majority of them do not have any clinical symptoms. However, the affected males have reduced visual acuity because of refractive errors or ocular oscillations. Ocular oscillations will result in unstable images on the retina, leading to sensory deprivation amblyopia^3^.

Mutations of the FERM domain containing 7 (*FRMD7*) gene have been documented to be responsible for the X-linked IN^4,5^. Up to date, approximately 110 mutations in the *FRMD7* have been identified in the patients with X-Linked IN, including missense, nonsense, and splicing mutations, as well as indel mutations^6^.

Conventionally, nystagmus is measured in terms of its amplitude, frequency and their product of intensity which is usually used to describe the severity of nystagmus^7–9^. Foveation period is another parameter to predict the visual function because it reflects the correlations of the fovea with the velocity and position of image on the retina^8^. Patients with foveation period usually have a good visual acuity at near even at distance. However, it is not clear whether the intensity of nystagmus and foveation period are determined by the genotype of *FRMD7*. In this study, we evaluated the difference of the ocular oscillations between patients with and without *FRMD7* mutation, and investigated the correlation of the ocular oscillations with different types of mutations.

## Methods

### Participants

Medical records were retrospectively reviewed for 56 pedigrees and 34 sporadic patients with IN in this study. Diagnosis of IN was based on the following criteria: (1) onset of nystagmus within 3–6 months after birth; (2) without disorders in the eye or visual pathway; (3) without abnormality in the nervous system or mental disorders. Ophthalmic examinations included slit lamp examination for the anterior segment of eye, ophthalmoscope and photography for fundus examinations. Fundus structure was examined by the optical coherence tomography (OCT) (Heidelberg Engineering, Germany). The best corrected visual acuity at near and distance were evaluated using the Snellen Charts under the conditions of monocular and binocular fixations. Refractive errors were examined by the equipment of the VS100-Welch Allyn. Ocular alignment and ocular movement were assessed at nine cardinal gaze positions. Binocular sensory status was evaluated with the Bagolini striated glasses at near and distance, and by stereoacuity assessment at near using the Titmus test (Stereo Optical Co., Inc., Chicago, IL).

Molecular diagnosis was performed for all patients and their family members under their informed consents. Informed consents were obtained from parents/Legal guardian of few minors in this study. This study complied with the tenets of the Declaration of Helsinki, and approved by the Ethics Committee of the Beijing Children’s Hospital.

### Molecular diagnosis

Three milliliters of peripheral venous blood were collected from each participant. Genomic DNA was extracted from the lymphocytes according to the standard protocol (Roche Biochemical, Inc). All coding exons and exon–intron boundaries of *FRMD7* (NG_012347 and NM_194277) were amplified by the polymerase chain reaction described previously^10^. The PCR products were extracted using the QIAquick Gel Extraction Kit (Qiagen, Valencia, CA). Direct sequencing was performed using the BigDye Terminator Cycle Sequencing v3.1 Kit on an ABI 3130 Genetic Analyzer (Applied Biosystems). Sequences were analyzed using the Seqman program in the DNASTAR software (DNASTAR Inc., Madison, WI). The variants were searched in databases including dbSNP151, EXAC, gnomAD 2.1, ClinVar and HGMD2021. Pathogenicity prediction scores were obtained for missense variants using SIFT, PolyPhen-2, MutationTaster and CADD, and further confirmed by modeling the 3-D structure of protein using the PyMOL program. Alterations affecting splicing were assessed using the SpliceAI program. Mutations were named following the nomenclature recommended by the Human Genomic Variation Society (HGVS). The pathogenicity of all variants in the *FRMD7* gene was assessed according to the ACMG/AMP guidelines^11^.

### Eye movement recording

The amplitude and frequency of nystagmus were recorded by the Eyelink 1000 Plus (SR Research Ltd., Canada). Subjects faced to the recording equipment at a distance of 60 cm to the screen and of 55 cm to the camera. They were required to sit down on a chair with their heads stabilized on the chin and forehead rest, without wearing glasses or contact lenses. EyeLink data was recorded at 1000 Hz. Eye movement recordings started with a calibration program in which a fixation target was displayed at 0°, horizontal ± 15°, and vertical ± 10° on a screen. After calibration and validation, eye movements were recorded with the program of Pupil-CR, and displayed as traces in the Plot View.

### Statistical analysis

Descriptive analysis was performed. Frequency (proportion) or mean +/− SD was reported for categorical or continuous variables, respectively. Analysis of Variance (ANOVA) was used to compare the difference among the patients with and without mutations. A *P* value of < 0.05 was considered statistically significant. No multiplicity adjustment was implemented. All statistical analyses were performed by R (version, 4.1.0).

## Results

### Mutation identification

**Figure 1:**
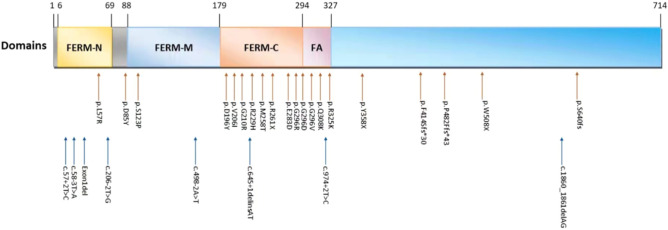
Schematic representation of 28 novel *FRMD7* mutations.

**Figure 2:**
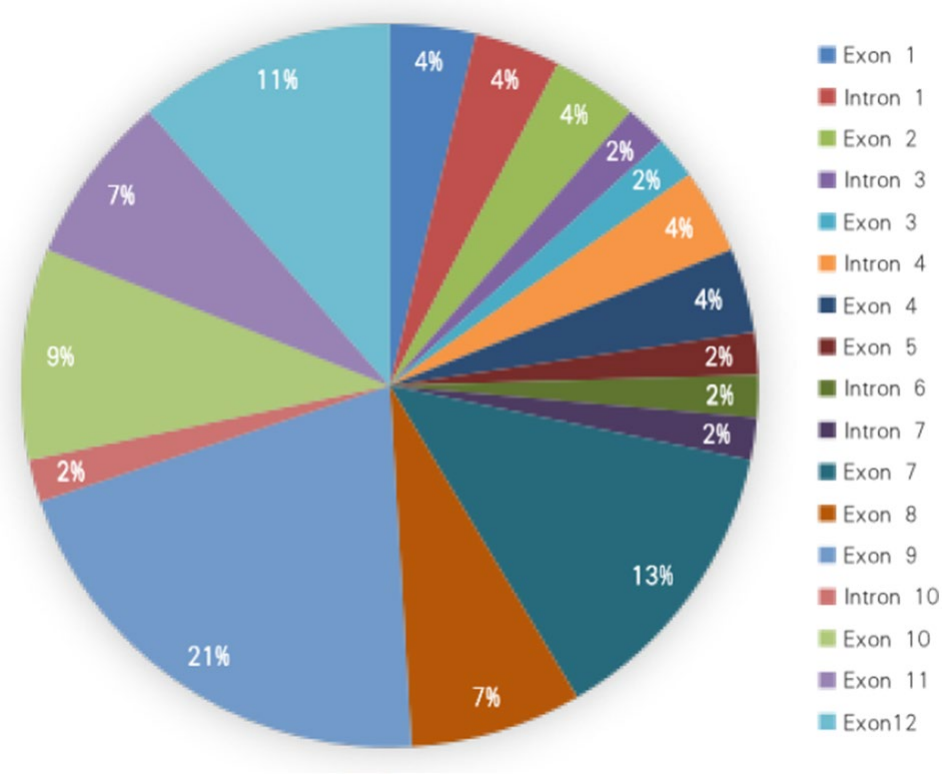
Distribution of 52 Mutations in the *FRMD7* gene.

Fifty-two mutations were detected in *FRMD7* gene from 56 pedigrees and 34 sporadic patients with IN, in which 28 were novel and 24 were previously reported mutations (Table 1 and Fig. 1). The types of 52 mutations included: 28 missense (14 novel), 6 nonsense (3 novel), 9 splicing (6 novel), 8 deletion (4 novel), and 1 small insertion (novel). They distributed into all 12 exons of *FRMD7*, where 41% (21/52) mutations clustered in exon 7, 8 and 9 (Fig. 2). All 56 pedigrees were detected to carry *FRMD7* mutations; however, because mutations of c.170T > G (p.L57R), c.623A > G (p.H208R), c.773T > C (p.M258T) and c.887G > C (p.G296R) were present in two or more non-consanguineous pedigrees, only 46 kinds of mutations were detected from 56 pedigrees. To examine the possibility of a common origin among those families carrying the same mutation, four SNPs of rs2180237, rs1569893, rs2748723, and rs2748724 in and around *FRMD7* were genotyped in the affected males. Haplotype analysis showed that mutations of c.170T > G (p.L57R), c.623A > G (p.H208R), c.773T > C (p.M258T) and c.887G > C (p.G296R) occurred independently in each family. Six of 52 mutations were detected in the sporadic patients.

**Table 1:** Mutations of *FRMD7* in 56 pedigrees and 34 sporadic patients with infantile nystagmus.

No	Source	Gend- er	age	Location	Mutation	Protein	Type	Report	BCVA (OD/OS)	ACMG classification	Evidence levels	SIFT	CADD	Polyphen2	SpliceAI
1	F1	M	7	Intron 1	c.57 + 1G > A		Splicing	PMID: 28656292	0.4/0.6	Pathogenic	PVS1, PS4, PM2	–	24	–	DL(0.96)
2	S1	M	6	Intron 1	c.57 + 2 T > C		Splicing	None	0.5/0.5	Likely pathogenic	PVS1, PM2	–	23.4	–	DL(0.92)
3	F2	M	9	Intron 1	c.58-3 T > A		Splicing	None	0.6/0.7	Uncertain	PM2	–	–	–	AL(0.78)
4	F3	M	5	Exon 1	Exon1		Deletion	None	0.6/0.6	Likely pathogenic	PVS1, PM2	–	–	–	
5	F4	M	4	Exon 2	c.70G > T	p.G24W	Missense	PMID: 18431453	0.4/0.5	Pathogenic	PS4, PM1, PM2, PM5, PP3	0	33	1	
6	F5	F	6	Exon 2	c.70G > A	p.G24R	Missense	PMID: 17013395	0.6/0.6	Pathogenic	PS4, PM1, PM2, PM5, PP3	0	29.6	1	
7	F6	F	17	Intron 3	c.162 + 2 T > C		Splicing	PMID: 28623544	0.4/0.5	Pathogenic	PVS1, PS4, PM2	–	24.5	–	DL(0.97)
8	F7	M	8	Exon 3	c.170 T > G	p.L57R	Missense	None	0.6/0.6	Uncertain	PM2, PP3	0	25.3	0.999	
9	F8	M	7	Exon 3	c.170 T > G	p.L57R	Missense	None	0.9/0.8	Uncertain	PM2, PP3	0	25.3	0.999	
10	F9	M	7	Intron 4	c.206-1G > A		Splicing	PMID: 28623544	0.4/0.4	Pathogenic	PVS1, PS4, PM2	–	25.3	–	AG(0.53) AL(0.75)
11	F10	M	6	Intron 4	c.206-2 T > G		Splicing	None	0.5/0.4	Likely pathogenic	PVS1, PM2	–	24.1	–	AG(1.00) AL(0.75)
12	F11	M	9	Exon 4	c.253G > T	p.D85Y	Missense	None	0.5/0.5	Uncertain	PM2, PP3	0.01	29.2	0.992	
13	F12	F	3	Exon 4	c.284G > T	p.R95M	Missense	PMID: 28623544	NA	Likely pathogenic	PS4, PM2, PP3	0	28.6	0.987	
14	F13	M	3	Exon 5	c.367 T > C	p.S123P	Missense	None	0.4/0.4	Uncertain	PM2, PP3	0.001	29	0.997	
15	F14	M	6	Intron 6	c.498-2A > T		Splicing	None	0.5/0.4	Likely pathogenic	PVS1, PM2	–	25.9	–	AG(0.95) AL(0.97)
16	F15	F	8	Exon 7	c.521A > T	p.D174V	Missense	PMID: 28623544	0.5/0.5	Likely pathogenic	PS4, PM2, PP3	0	27.8	0.967	
17	S2	M	9	Exon 7	c.580G > A	p.A194T	Missense	PMID: 32446246	0.8/0.7	Uncertain	PM1, PM5, PP3	0.004	34	0.998	
18	F16	F	6	Exon 7	c.586G > T	p.D196Y	Missense	None	0.6/0.4	Uncertain	PM2, PM5, PP3	0	30	0.999	
19	F17	F	13	Exon 7	c.601C > T	p.Q201X	Nonsense	PMID: 17013395	0.7/0.7	Pathogenic	PVS1, PS4, PM2	–	37	–	
20	F18	M	8	Exon 7	c.616G > A	p.V206I	Missense	None	0.5/0.6	Uncertain	PM2	0.005	29.2	0.994	
21	F19	F	11	Exon 7	c.623A > G	p.H208R	Missense	PMID: 21365021	0.8/0.8	Likely pathogenic	PS4, PM2, PP3	0.001	24.7	0.994	
22	F20	M	7	Exon 7	c.623A > G	p.H208R	Missense	PMID: 21365021	0.4/0.3	Likely pathogenic	PS4, PM2, PP3	0.001	24.7	0.994	
23	F21	M	9	Exon 7	c.628G > C	p.G210R	Missense	None	0.5/0.6	Uncertain	PM2, PP3	0	33	1	
24	F22	M	9	Intron 7	c.645 + 1delinsAT		Splicing	None	0.4/0.4	Uncertain	PM2	–	–	–	DL(0.98)
25	F23	F	5	Exon 8	c.685C > T	p.R229C	Missense	PMID: 17768376	0.5/0.5	Pathogenic	PS4, PM1, PM2, PM5, PP3	0.001	34	0.999	
26	S3	F	2	Exon 8	c.686G > A	p.R229H	Missense	None	NA	Likely pathogenic	PM1, PM2, PM5, PP3	0.001	35	0.999	
27	F24	M	9	Exon 8	c.689-690delAG	p.S232Ffs*2	Deletion	PMID: 18431453	0.7/0.7	Likely pathogenic	PVS1, PM2	–	–	–	
28	F25	M	12	Exon 8	c.694_695delAG	p.S232FfsX233	Deletion	PMID: 18431453	0.9/0.9	Pathogenic	PVS1, PS4, PM2	–	–	–	
29	F26	M	7	Exon 9	c.773 T > C	p.M258T	Missense	None	0.5/0.4	Likely pathogenic	PM1, PM2, PM5, PP3	0	26.2	0.974	
30	F27	M	9	Exon 9	c.773 T > C	p.M258T	Missense	None	0.7/0.7	Likely pathogenic	PM1, PM2, PM5, PP3	0	26.2	0.974	
31	F28	M	8	Exon 9	c.766 T > A	p.F256I	Missense	PMID: 28623544	0.4/0.4	Likely pathogenic	PS4, PM2, PP3	0	29.4	0.996	
32	S4	M	4	Exon 9	c.781C > T	p.R261X	Nonsense	None	0.5/0.6	Likely pathogenic	PVS1, PM2	–	38	–	
33	F29	F	7	Exon 9	c.782G > A	p.R261Q	Missense	PMID: 18431453	0.7/0.7	Pathogenic	PS4, PM1, PM2, PM5, PP3	0.002	34	0.996	
34	F30	M	5	Exon 9	c.782G > A	p.R261Q	Missense	PMID: 18431453	0.5/0.5	Pathogenic	PS4, PM1, PM2, PM5, PP3	0.002	34	0.996	
35	F31	M	9	Exon 9	c.811 T > C	p.C271R	Missense	PMID: 28623544	0.6/0.5	Pathogenic	PS4, PM1, PM2, PM5, PP3	0	26.7	0.998	
36	F32	F	7	Exon 9	c.812G > T	p.C271F	Missense	PMID: 18431453	1.0/1.0	Pathogenic	PS4, PM1, PM2, PM5, PP3	0	33	0.997	
37	F33	M	8	Exon 9	c.837G > C	p.R279S	Missense	PMID: 18685727	0.8/0.7	Likely pathogenic	PS4, PM2, PP3	0	25.1	0.996	
38	F34	M	6	Exon 9	c.849G > C	p.E283D	Missense	None	0.5/0.6	Uncertain	PM2	0.017	26.2	0.99	
39	F35	F	8	Exon 9	c.887G > C	p.G296R	Missense	None	0.4/0.4	Uncertain	PM2, PM5, PP3	0.002	28.2	0.999	
40	F36	F	11	Exon 9	c.887G > C	p.G296R	Missense	None	0.7/0.6	Uncertain	PM2, PM5, PP3	0.002	28.2	0.999	
41	F37	M	9	Exon 9	c.887G > C	p.G296R	Missense	None	0.5/0.5	Uncertain	PM2, PM5, PP3	0.002	28.2	0.999	
42	F38	M	5	Exon 9	c.887G > A	p.G296D	Missense	None	0.5/0.7	Uncertain	PM2, PM5, PP3	0	32	1	
43	S5	F	4	Exon 9	c.887G > T	p.G296V	Missense	None	0.6/0.6	Uncertain	PM2, PM5, PP3	0	33	1	
44	F39	M	7	Exon 10	c.910C > T	p.R304X	Nonsense	PMID: 18431453	0.4/0.6	Pathogenic	PVS1, PS4, PM2	–	37	–	
45	F40	M	3	Exon 10	c.922C > A	p.Q308K	Missense	None	NA	Uncertain	PM2, PP3	0.002	28.5	0.991	
46	F41	M	9	Exon 10	c.973A > G	p.R325G	Missense	PMID: 28623544	0.4/0.5	Uncertain	PM2, PM5, PP3	0	24	0.359	
47	F42	F	2	Exon 10	c.974G > A	p.R325K	Missense	None	NA	Uncertain	PM2, PM5	0.089	25.1	0.269	
48	S6	M	20	Intron 10	c.974 + 2 T > C		Splicing	None	0.4/0.4	Likely pathogenic	PVS1, PM2	–	24.5	–	DL(0.77)
49	F43	M	5	Exon 11	c.980_987delATTACCCAinsCCAA	p.H327Pfs *27	Deletion	PMID: 23733424	0.4/0.4	Pathogenic	PVS1, PS4, PM2	–	–	–	
50	F44	M	6	Exon 11	c.986C > A	p.P329Q	Missense	PMID: 23733424	0.6/0.7	Likely pathogenic	PS4, PM2, BP4	0.37	23.9	0.037	
51	F45	M	7	Exon 11	c.999delT	p.H333fs*22	Deletion	PMID: 28623544	0.5/0.5	Pathogenic	PVS1, PS4, PM2	–	–	–	
52	F46	F	6	Exon 11	c.1003C > T	p.R335X	Nonsense	PMID: 17013395	0.7/0.7	Pathogenic	PVS1, PS4	–	39	–	
53	F47	M	3	Exon12	c.1074T > G	p.Y358X	Nonsense	None	NA	Likely pathogenic	PVS1, PM2	–	36	–	
54	F48	F	10	Exon12	c.1241delT	p.F414Sfs*30	Deletion	None	0.8/0.8	Likely pathogenic	PVS1, PM2	–	–	–	
55	F49	M	5	Exon12	c.1442_1443insAT	p.P482Ffs*43	Insertion	None	0.5/0.6	Likely pathogenic	PVS1, PM2	–	–	–	
56	F50	M	6	Exon12	c.1523G > A	p.W508X	Nonsense	None	0.8/0.8	Likely pathogenic	PVS1, PM2	–	33	–	
57	F51	F	8	Exon12	c.1860_1861delAG	p.D621Pfs*27	Deletion	None	0.4/0.6	Likely pathogenic	PVS1, PM2	–	–	–	
58	F52	M	7	Exon12	c.1918delA	p.S640fs	Deletion	None	0.6/0.7	Likely pathogenic	PVS1, PM2	–	–	–

**Figure 3:**
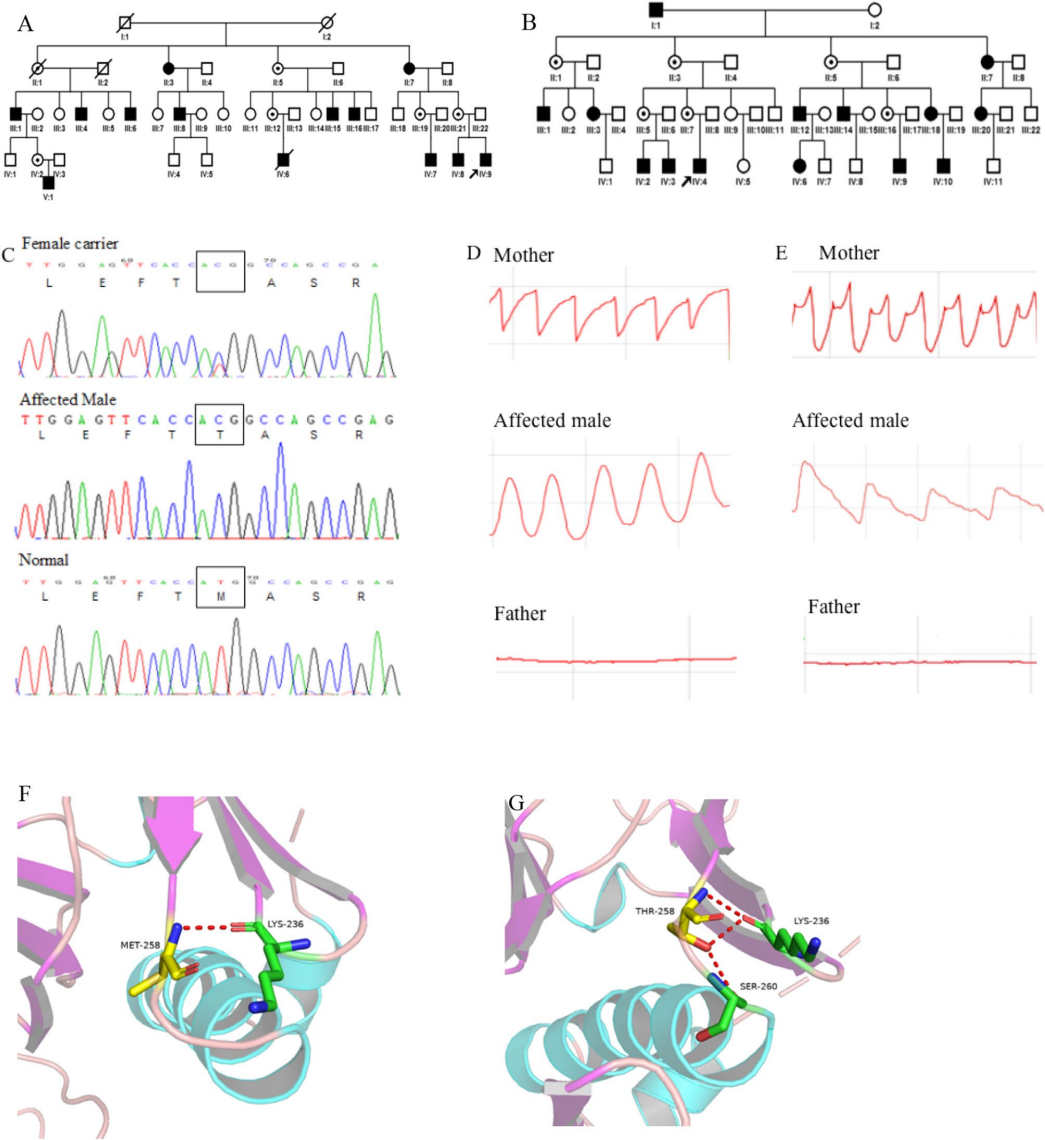
*FRMD7* mutation and waveforms recorded in two families. The probands in families **A** and **B** (denoted by the black arrows) carried the same mutation of c.773 T > C (p.M258T) in exon 9 (**C**). Nystagmus was recorded as horizontal pendular waveforms from the proband (**D**, middle panel), and as decelerating exponential slow phase jerk waveforms from his mother (**D**, upper panel) in the Family **A**. As a control, his father had normal eye movement recordings (**D**, lower panel). The proband in Family **B** showed horizontal decelerating exponential slow phase jerk waveforms (**E**, middle panel), and his mother had horizontal dual jerk waveforms (**E**, upper panel), compared with his father (**E**, lower panel). A 3-D model construction showed a wild-type nonpolar amino acid of Methionine (**F**) was replaced by a charged and polar amino acid of Threonine (**G**), which would damage the stability of the protein structure and function.

All novel missense mutations identified in this study were predicted to be deleterious to the protein function and structure through online program analysis by using SIFT, PolyPhen-2, MutationTaster, CADD and PyMOL. The calculated scores for all mutations using the programs of SIFT, Polyphen2, CADD and Splice AI, as well as the ACMG classification, were listed in Table 1. A summarized description of PyMol results was listed in Table 2. As an example, the structure damage by M258T was shown in Fig. 3, and others were not shown here but may be supplied if requested.

**Table 2:** Description of PyMol structure prediction for 14 novel missense mutations.

No	Mutation	Protein	PyMol
1	c.170 T > G	p.L57R	Wild-type L57 is located in ß -folding region. The molecular quantity of mutant-type R57 is increased, the polarity is changed from non-polar to positively charged, and from neutral to alkaline. Mutant-type R57 will bring the strong basic amino acid residue I63 into the hydrophobic region, and destroy protein stability
2	c.253G > T	p.D85Y	The molecular quantity of mutant-type Y85 is increased, the polarity is changed from negatively charged to uncharged. Y85 loses interaction with G87
3	c.367T > C	p.S123P	Wild-type S123 is located in the a-helix region. The molecular quantity of mutant-type P123 is increased, the polarity is changed from uncharged polar to non-polar. P123 loses interaction with A119, L120, and I158
4	c.586G > T	p.D196Y	The molecular quantity of mutant-type Y196 is increased, the polarity is changed from negatively charged to uncharged. Y196 loses interaction with S195 and E198
5	c.616G > A	p.V206I	The molecular quantity of mutant-type I206 is increased
6	c.628G > C	p.G210R	The molecular quantity of mutant-type R210 is increased, the polarity is changed from uncharged to positively charged
7	c.686G > A	p.R229H	Wild-type R229 is replaced by mutant-type H229, which interacts with K241 by two hydrogen bonds. The molecular quantity of mutant H229 is decreased
8	c.773 T > C	p.M258T	Wild-type M258 interacts with amino acid residues K236 and P193. The molecular quantity of mutant-type T258 is decreased, the polarity is changed from non-polar to uncharged polar. Mutant-type T258 loses interaction with P193 and increases interaction with S260. S260 is a phosphorylation site. T258 affects FRMD7 regulation through phosphorylation
9	c.849G > C	p.E283D	The molecular quantity of mutant-type D283 is decreased. D283 increases interaction with K285
10	c.887G > C	p.G296R	Wild-type G296 and the adjacent amino acid residue S294 form the ß -folded region. The molecular quantity of mutant-type R296 is increased, the polarity is changed from uncharged polar to positively charged. R296 increases interaction with S298
11	c.887G > A	p.G296D	Wild-type G296 and the adjacent amino acid residue S294 form the ß -folded region. The molecular quantity of mutant-type D296 is increased, the polarity is changed from uncharged to negatively charged.D296 loses interaction with S294 and increases interaction with S297
12	c.887G > T	p.G296V	Wild-type G296 and the adjacent amino acid residue S294 form the ß -folded region. The molecular quantity of mutant-type V296 is increased, the polarity is changed from uncharged polar to non-polar. V296 loses interaction with S294
13	c.922C > A	p.Q308K	The molecular quantity of mutant-type K308 is minor increased, the polarity is changed from uncharged to positively charged
14	c.974G > A	p.R325K	The molecular quantity of mutant-type K325 is decreased

### Comparisons of parameters

**Figure 4:**
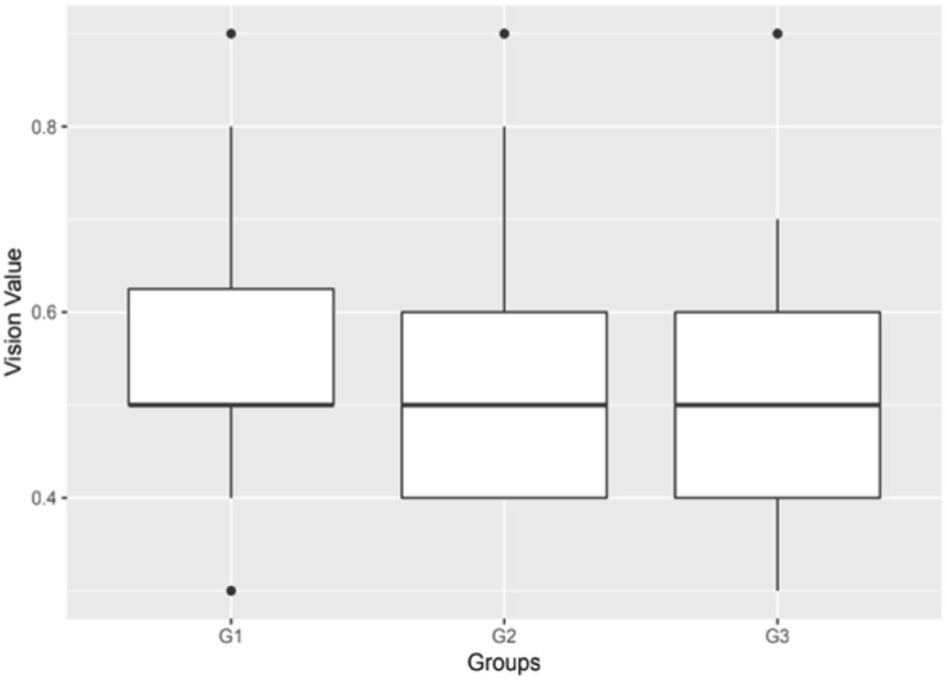
Box plots showing median levels of the BCVA in three groups of patients.

Affected males with or without *FRMD7* mutations were collected to compare the difference between the best corrected visual acuity (BCVA) and nystagmus intensity because the affected males were hemizygote and carried only one affected allele on the X-chromosome. For comparison, the affected males were categorized into three subgroups. Group 1 (G1) consisted of 20 affected males with missense mutations; Group 2 (G2) included 18 affected males with premature translation termination; and Group 3 (G3) was composed of 19 affected males without *FRMD7* mutations. The BCVA was evaluated for all patients in three groups. However, eye movement recording was obtained 14 in G1, 13 in G2 and 13 in G3. The mean of BCVA was 0.56 ± 0.14 in G1, 0.55 ± 0.15 in G2, and 0.52 ± 0.15 in G3 (Fig. 4). There were no significant differences in BCVA among these three groups (P = 0.474, P > 0.05).

The waveforms of nystagmus were varied as pendular, pendular with foveating saccades, jerk, jerk with extended foveation, as well as dual jerk waveforms recorded in our patients. No specific waveforms were associated with a specific mutant type. Even within a pedigree, waveforms were varied in the patients and their relatives, such as in a pedigree with a mutation of c.773T > C (p.M258T) (Fig. 3D,E).

**Figure 5:**
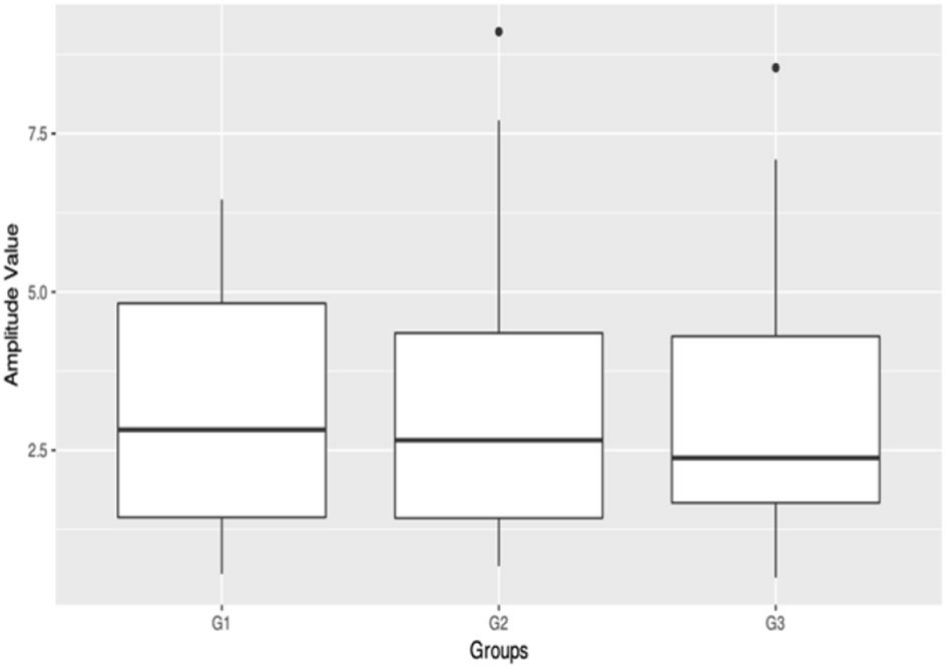
Box plots showing median levels of the amplitude in three groups of patients.

**Figure 6:**
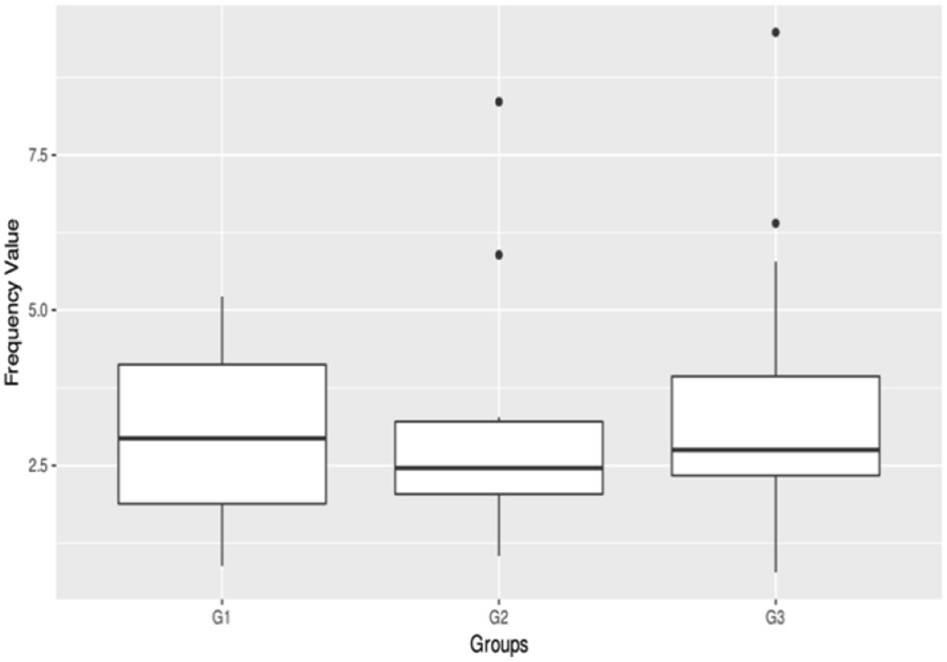
Box plots showing median levels of the frequency in three groups of patients.

Eye movement recordings showed that the amplitude of oscillation was on the average of 3.20° ± 1.93°in G1, 3.44° ± 2.53° in G2, and 3.29° ± 2.39°in G3 at the primary gaze position (Figs. 5, 6). There were no significant differences among these three groups in the amplitude (*P* = 0.967, *P* > 0.05). While the frequency of oscillation was a mean of 2.97 Hz ± 1.35 Hz in G1, 2.99 Hz ± 1.92 Hz in G2, and 3.64 Hz ± 2.24 Hz in G3 at the primary gaze position. There were no significant differences among these three groups in the frequency (*P* = 0.609, *P* > 0.05).

## Discussion

In this study, we found 52 mutations in *FRMD7* from 56 Chinese pedigrees and 34 sporadic patients with IN, including 28 novel and 24 known mutations. To our knowledge, the number of mutations detected in this study is the largest in a series of reports about investigating *FRMD7* mutations in the patients with IN. The mutation detection rate is higher in the X-linked pedigrees (46/56) than that in the sporadic patients (6/34). We suggest that the lower detection rate in the sporadic patients could be related to uncertainties in the inheritance modes, which might include new dominant or recessive inheritance patterns. This would be consistent with previous findings, which show that the X-linked mode accounts for 90% IN patients with family history, the autosomal dominant mode accounts for about 10%, and the autosomal recessive pattern is exceedingly rare.

Recurrent mutations and mutations at the same codon are also found in the patients in different families. However, recurrent mutations have been proven to be independently from a different chromosomal background rather than a common founder through haplotype analysis. As shown in Table 2, c.70G > T (p.G24W) and c.70G > A (p.G24R) represent a change from a highly conserved small neutral amino acid to larger hydrophobic polar and positively charged amino acids, respectively. These are not conservative in terms of their physical characteristics as estimated using a Grantham formula^12^. Similarly, c.887G > C (p.G296R), c.887G > A p.G296D), and c.887G > T (p.G296V) represent changes from a highly conserved very small, neutral amino acid to larger positively charged, negatively charged hydrophilic, and aliphatic hydrophobic amino acids, respectively, also distant on the Grantham chart. These would be expected to disrupt the local environment and structure of the FRMD7 protein with subsequent interference with its function.

*FRMD7* encodes a protein with 714 amino acids belonging to the FERM-domain family members due to four proteins of the protein 4.1, ezrin, radixin and moesin in its structure^13^. As in most other FERM-domain family members, the highly conserved N-terminus consists of three domains of FERM-N, FERM-M, and FERM-C which are responsible for localizing the proteins to the plasma membrane^14,15^. The FERM-adjacent (FA) domain is located in the middle of the N-terminus and C-terminus of FRMD7 protein, playing a role in regulation of protein function through modifications such as phosphorylation. The C-terminus of FRMD7 has no significant homology with other proteins^15^. FRMD7 is highly expressed in the retina and midbrain where the center of ocular movement is located, and plays an important role in neurite development as well as in the control of eye movement and gaze stability^5,13^.

**Figure 7:**
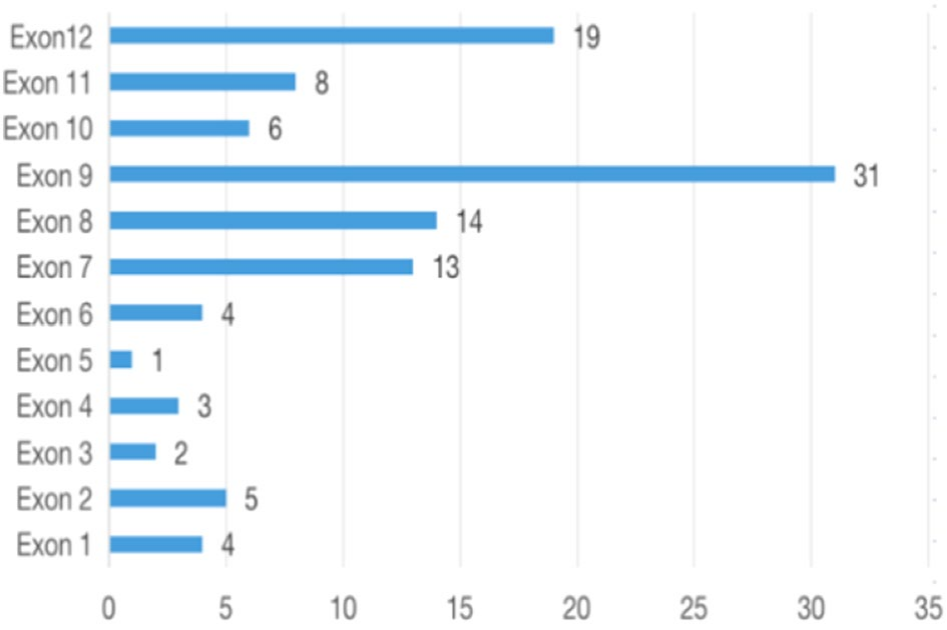
Distribution of *FRMD7* mutations within exons identified in this study and previous reports.

Mutations of *FRMD7* were reported to cluster highly in the FERM and FA domains, and to present frequently in exon 2, exon 8 and 9 in the previous study^16^. In our study, 65% (34/52) mutations were found to present in the FERM and FA domains, which further supported that FERM and FA domains were the hotspot of mutation. The frequency of mutation was highest in exon 9 (21%, 11/52), followed by exon7 (13%, 7/52) (Fig. 2). We reviewed all 110 mutations reported in the Human Genome Mutation Database, and reanalyzed these mutations with 28 novel detected mutations in this study. Similarly, the frequency of mutation is highest in exon 9 (22%, 31/138), followed by exon 12 (19/138), exon 8 (10%, 14/138), and exon 7 (9%, 13/138) (Fig. 7). In total, 41% (57/138) mutations are highly clustered in the exon 7, 8 and 9. However, only 3% (5/ 138) mutations are detected from exon 2, inconsistent with the previous report that exon 2 is a mutation-rich area^16^.

Structurally, the FERM-C domain is the third domain within the FERM domain, and constituted mainly by amino acids encoded from exon 7–9. This domain has many specific protein binding sites, playing important roles in linking the membrane with cytoskeleton as well as transmitting signals^17–19^. Recently, FRMD 7 has been found to bind to CASK and GABRA2. As a member of the membrane-associated guanylate kinase (MAGUK) family, CASK is found to be involved in regulation of neurite outgrowth and formation of dendritic spines. *FRMD7* mutations would disrupt the interaction with CASK, impairing CASK-induced neurite formation and affecting neural and retinal development^13^. Clinically, hypoplasia of fovea and optic nerve have been detected from patients with *FRMD7* mutations^20^. Thus, one of possible mechanisms of nystagmus could be related to the arrested neural and retinal development during the early embryonic phase^21^. Another possible mechanism of nystagmus could be related to the signal transmission. The inhibitory neurotransmitter of GABRA2 plays a significant role in transmitting signals between the starburst amacrine cells (SACs) and the directional selective ganglion cells (DSGCs), as well as between SACs and SACs. *FRMD7* mutations would prevent the inhibitory signal transmitting from SACs to DSGCs through disruption of the interaction with GABRA2, leading to the loss of optokinetic reflex (OKR) and resulting in INS^22^. Thus, it is reasonable to speculate that FRMD7 protein could function as a “bridge” in neural and retinal development, as well as signal transmission through “binding” with other proteins.

**Figure 8:**
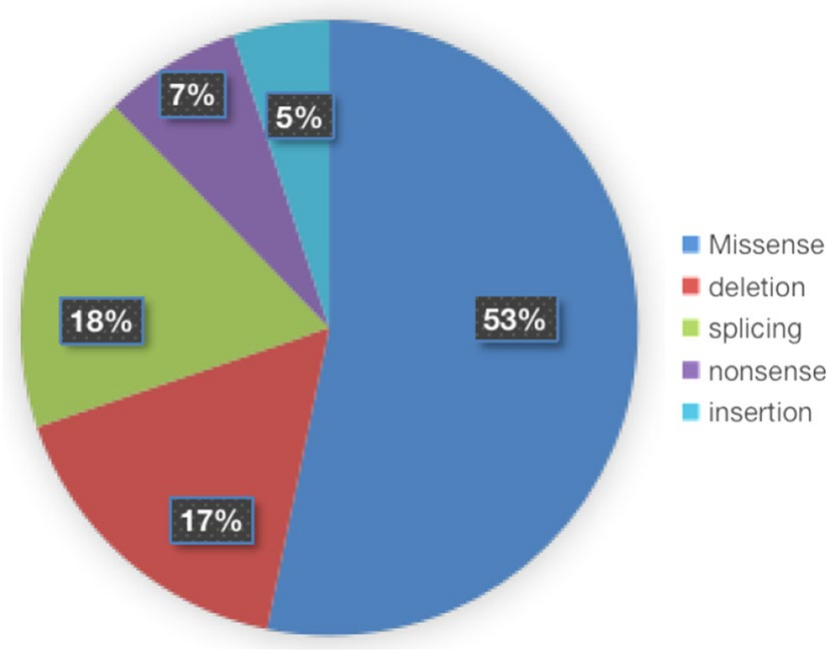
Proportions of *FRMD7* mutation types identified in this study and reported recently.

To date, 73 missense mutations have been detected from patients with IN, including 14 novel mutations detected in this study, accounting for 53% (73/138) of IN-associated mutations (Fig. 8). Others are nonsense, splicing and indel mutations which would produce a premature terminate codon and generate either a truncated protein or an abnormal mRNA that would be degraded due to the nonsense-mediated decay mechanism. The previous study showed that the severity of IN was highly associated with the amount and the location of the expressed protein^13^. Missense mutants with lower expression in the cytoplasm or localized aberrantly to the nucleus would produce a severe clinical feature due to the dominant-negative mechanism, while mutants caused by premature terminated translation would be possible to produce a much severer clinical phenotype, because mutants would be restricted to the nucleus^13^.

Our data does not support the correlation between the genotype and the phenotype in IN. According to the previous study, the affected males carrying with premature terminated translation would have much severer clinical features than those males who carry with missense mutations. However, there is no statistical difference between them on their corrected visual acuity, and on nystagmus amplitude and frequency. As far as the visual acuity is concerned, even though among the patients in a pedigree carrying with a common mutation, patients have variable visual acuity. For example, in the pedigree with a missense mutation of c.812G > T (p.C271F), patients had variable visual acuity from 0.2 to 1.0, while in the pedigree carrying with mutation of c.689-690delAG (p.S232Ffs*2), the visual acuity varied from 0.4 to 1.0 in the patients. Because the female carriers usually do not have symptoms, we think that loss-of-function could be the common molecular mechanism for the X-linked infantile nystagmus, no matter what kinds of mutations they carry.

## Conclusions

We identify 28 novel mutations in the *FRMD7* gene which would further expand the spectrum of *FRMD7* mutations. The mutation hotspot is located at the FERM-C domain and is clustered in exon 7–9. The intensity of oscillations is not determined by the types of *FRMD7* gene mutations, and not determined by absence or presence of mutations. Loss-of-function could be the common molecular mechanism for the X-linked infantile nystagmus.

## Supplementary Information

Supplementary Information.
