# Supplementary figures and images for: Correlations of FRMD7 gene mutations with ocular oscillations
Source: Sci Rep. 2022 Jun 15;12:9914. doi: 10.1038/s41598-022-14144-7 (PMC9200781; doi:10.1038/s41598-022-14144-7)

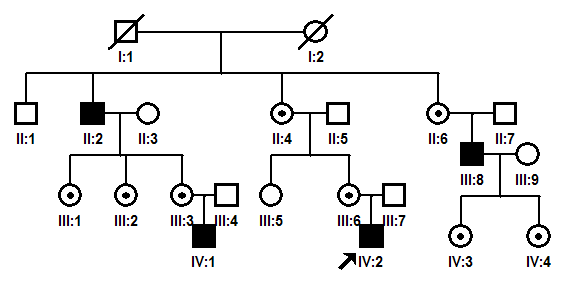

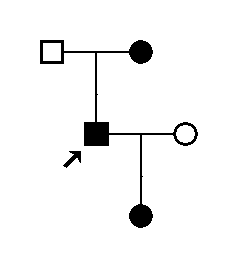


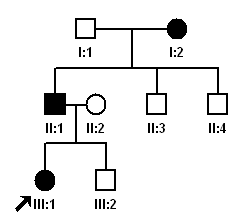

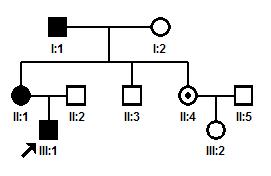


The four pedigrees of IN without mutation detected.

Supplement: Supplementary file 1 — Supplementary Information. [file 41598_2022_14144_MOESM1_ESM.docx]
